# Supplementary material for: Stochastic step-wise feature selection for Exponential Random Graph Models (ERGMs)
Source: PLoS One. 2024 Dec 17;19(12):e0314557. doi: 10.1371/journal.pone.0314557 (PMC11651600; doi:10.1371/journal.pone.0314557)
Supplement: Attachment — Submitted filename: report.pdf [file pone.0314557.s002.pdf]

Stochastic Stepwise Selection for Exponential Random Graph Models (ERGMs)

by Helal El-Zaatari, Fei Yu and Micahel R. Kosorok

submitted to Computational Statistics

Referee report

The paper under revision addresses the problem of endogenous variable selection in Exponential Random Graph Models (ERGMs) using a novel step-wise variable selection process.

In the initial step of the method, an initial set of endogenous variables is determined, including commonly used variables such as k-stars and triangles. During the selection process, a forward algorithm based on the AIC is employed, where the baseline model is an ERGM that solely counts the number of edges in the graph.

The method shows promise. However, the paper's structure and the numerous inconsistencies in explaining the method and the manuscript's writing make it challenging to comprehend. It would be beneficial to enhance the clarity and coherence of the paper's writing. I kindly suggest that the authors consider making improvements to the paper and resubmit it for further consideration.

I also recommend that the authors include a simulation section in the manuscript. Simulating an exponential model with specific variables and subsequently verifying whether the proposed method correctly selects them would certainly enhance the paper.

Concerning the manuscript itself, below I comment on some more specific aspects.

1. In the first paragraph of page 3, the sentence "Inspired by generalized linear models, exponential random graphs model the probability of tie formation."appeared twice consecutively.

2. Equation (1) is not accurate. $Y_{i,j}$ represents the presence or absence of an edge between vertices i and j, while $\mathbf{Y}$ represents the entire network. The formulation of ERGMs models the probability of observing a network $\mathbf{Y}$, not the probability of an edge between a pair of vertices. It is also important to standardize the notation using y or $\mathbf{y}$. Equation (1) does not specify what $s(y)$ represents.

3. At the end of page 3, there is a typo: "...demands the selection of an input k N."

4. On page 4, it is stated that "The identification of N_k plays a pivotal role in dictating the size of the initial set of endogenous variables denoted by S_N. Applying this method to an observed network results in a list of N candidate endogenous variables, $\mathcal{S}_N$". However, earlier the authors mentioned the graph has N nodes; I do not see the explanation for why the size of the list of candidate endogenous variables, $\mathcal{S}_N$, matches the number of nodes in the graph.

5. In Algorithm 1, steps 1 and 2, it would be clearer to use a different notation, since j is used to indicate graph nodes. I recommend changing the notation for clarity.

6. On page 5, in the paragraph after Category 3, there is a typo: "...fit univariate ERGMs for each element in s $s_i(y)$."

7. Similar to Comments (2) and (4), the formulation of Equation (2) is confusing. It suggests that the exogenous variable $s_i(y)$ depends on node i, as it represents the probability of tie formation between i and j.

8. Again, the probability in step (3) of Algorithm 2 is confusing and inaccurate.

9. What is the rationale behind choosing 4 as a lower bound in step (4) of Algorithm 3?

10. The organization of Section 1.3 is confusing. It starts by presenting Algorithm 3 based on network motifs, and then in Subsection 1.3.1, a network motif is explained.

11. I do not understand the meaning of this sentence: "Notably, for networks with fewer than 30 nodes, such as the Florentine marriage network, the algorithm consistently proposed fitting ERGMs."What were the conclusions drawn using the proposed method?

12. Again, the legend of Figure 2 is mathematically inconsistent.

13. In the main text, the authors pointed out thirteen exogenous variables widely used for ERGMs. However, in the real data analysis, it was reduced only to stars and triangles.

14. In general, it is important to clarify how the AIC is computed.
