# Supplementary material for: Stochastic step-wise feature selection for Exponential Random Graph Models (ERGMs)
Source: PLoS One. 2024 Dec 17;19(12):e0314557. doi: 10.1371/journal.pone.0314557 (PMC11651600; doi:10.1371/journal.pone.0314557)
Supplement: Attachment — Submitted filename: Comments.pdf [file pone.0314557.s003.pdf]

Stochastic Step-wise Feature Selection for Exponential Random Graph Models (ERGMs)

June 25, 2024

1 Comments

- In the section "Definition of ERGMs" (pages 2 and 3), the probabilistic model should be better defined. In Equation 1, the term $\mathbf{s}(\mathbf{y})$ (the vector of endogenous variables) is not defined along the text. Further, is it modeled the joint probability $P_\theta(\mathbf{Y} = \mathbf{y}|\mathbf{X} = \mathbf{x})$ or the marginal probability $P_\theta(Y_{ij} = 1|\mathbf{X} = \mathbf{x})$, as it is in Equation 1?
- It would be interesting a better explanation about endogenous (depend only on $\mathbf{y}$) and exogenous variables (depend on $(\mathbf{y},\mathbf{x})$). Since the focus is on endogenous variables selection, maybe the definitions, examples and/or interpretations of some endogenous variables such as kstar, triangle, etc should be added.
- In page 2, the sentence "Inspired by generalized linear models..." is written twice.
- The equations should be cited along the text. For example, in page 5, the term "... as shown below" should be changed by "...as shown in Equation 2".
- The notation N is used as the number of nodes but also the number of endogenous variables after the application of Algorithm 1. Maybe it is better to change one of them to avoid misunderstanding.
- How does the procedure deal with overfitting? Does the Algorithm 3 try to avoid it in any way? In the same algorithm, does the threshold equals to 4 of step 4 (model exclusion) have any empirical or theoretical justification?
- In the section of "Numerical Studies", it would be interesting to have some discussion or results about the application of Algorithm 1 in the networks. Maybe some table or comments about the obtained upper bounds of the endogenous variables (kstar, esp, etc).
