# Supplementary material for: Stochastic step-wise feature selection for Exponential Random Graph Models (ERGMs)
Source: PLoS One. 2024 Dec 17;19(12):e0314557. doi: 10.1371/journal.pone.0314557 (PMC11651600; doi:10.1371/journal.pone.0314557)
Supplement: Attachment — Submitted filename: Author_response_to_review_comments.pdf [file pone.0314557.s004.pdf]

Authors' Response to Reviews of

Stochastic Stepwise Feature Selection for Exponential Random Graph Models (ERGMs)

Helal El-Zaatari
PLOS ONE,

RC: ***Reviewers' Comment***, AR: Authors' Response, ☐ Manuscript Text

1. Reviewer #1

RC: ***In the first paragraph of page 3, the sentence "Inspired by generalized linear models, exponential random graphs model the probability of tie formation." appeared twice consecutively.***

AR: The extra sentence was deleted.

RC: ***Equation (1) is not accurate. The formulation of ERGMs models the probability of observing a network Y, not the probability of an edge between a pair of vertices. It is also important to standardize the notation using y or y. Equation (1) does not specify what s(y) represents***

AR: The definition was modified and s(y) was specified according to the following paper "Highly Scalable Maximum Likelihood and Conjugate Bayesian Inference for ERGMs on Graph Sets with Equivalent Vertices" which was published in PLOS ONE in 2022. Below is the equation and the paragraph.

$$P_\theta(Y = \boldsymbol{y}|\boldsymbol{\theta}, \boldsymbol{x}) = \psi(\boldsymbol{\theta}_1, \boldsymbol{\theta}_2)exp\{\boldsymbol{\theta}_1^T s(\boldsymbol{y}) + \boldsymbol{\theta}_2^T g(\boldsymbol{y}, \boldsymbol{x})\} \quad (1)$$

The network with n nodes is represented via an adjacency matrix Y on support $\mathcal{Y}_n$ and $\boldsymbol{y} \in \mathcal{Y}_n$. The vector of endogenous variables is represented via $s(\boldsymbol{y})$ where $s : \mathcal{Y}_n \longrightarrow \mathbb{R}^p$ and the vector of exogenous variables is represented via $g(\boldsymbol{y})$ where $g : \mathcal{Y}_n \longrightarrow \mathbb{R}^q$. The natural numbers $p, q \in \mathbb{N}$ depend on the number of endogenous and exogenous variable chosen.

RC: ***. At the end of page 3, there is a typo:***

AR: typo was fixed and is now:

"and demands the selection of an input $k \in \mathbb{N}$"

RC: ***On page 4, it is stated that "The identification of N_k plays a pivotal role in dictating the size of the initial set of endogenous variables denoted by $\mathcal{S}_N$. Applying this method to an observed network results in a list of N candidate endogenous variables, $\mathcal{S}_N$ ". However, earlier the authors mentioned the graph has N nodes; I do not see the explanation for why the size of the list of candidate endogenous variables, SN , matches the number of nodes in the graph.***

AR: The number of endogenous variables was changed to M instead of N to avoid confusion across the paper.

"Addressing the impracticality of considering an infinite yet countable number of endogenous variables, our approach involves sequentially fitting uni-variate ERGMs. Starting from $k = 1$ and progressing until we reach a specific cutoff point, $k = N_k$. This cutoff, N_k is determined when we achieve three or more consecutive parameter estimates reaching infinity. Beyond N_k, further consideration of endogenous variables becomes impractical, as they lead to coefficient estimates of negative infinity. The identification of N_k plays a pivotal role in dictating the size of the initial set of endogenous variables. Applying this method to an observed network results in a set of M candidate endogenous variables, $\mathcal{S}_M$, with the set's size determined by the upper bounds of variables like dsp, esp, nsp, kstar and the remaining nine endogenous variables."

RC: ***In Algorithm 1, steps 1 and 2, it would be clearer to use a different notation, since j is used to indicate graph nodes. I recommend changing the notation for clarity***

AR: The notation was changed from j to l to avoid confusion.

RC: ***On page 5, in the paragraph after Category 3, there is a typo:***

AR: typo was fixed to become: "We systematically fit uni-variate ERGMs for each element in s $s_i(\boldsymbol{y}) \in \mathcal{S}_M$ for $i \in \{1, \dots, M\}$ as shown in equation (2). "

RC: ***Similar to Comments (2) and (4), the formulation of Equation (2) is confusing***

AR: The equation was modified to match equation (1)

RC: ***Again, the probability in step (3) of Algorithm 2 is confusing and inaccurate***

AR: The probability of step (3) of Alogirhtm 2 was modified to match equation (1)

RC: ***. What is the rationale behind choosing 4 as a lower bound in step (4) of Algorithm 3***

AR: The main purpose of algorithm 3 is to avoid ERGM degeneracy which is characterized by excess generation of edges. The number 4 is a tuning parameter and was found empirically. It means that on average this ERGM produced four times the amount of edges than the observed network (indicating degeneracy). One can increase this number but will run the risk of obtaining degenerate ERGMs.

RC: ***The organization of Section 1.3 is confusing. It starts by presenting Algorithm 3 based on network motifs, and then in Subsection 1.3.1, a network motif is explained.***

AR: The network motif section is now presented before algorithm 3 to avoid confusion.

RC: ***I do not understand the meaning of this sentence: "Notably, for networks with fewer than 30 nodes, such as the Florentine marriage network, the algorithm consistently proposed fitting ERGMs."What were the conclusions drawn using the proposed method?***

AR: This sentence was deleted.

RC: ***Again, the legend of Figure 2 is mathematically inconsistent***

AR: The equation was modified to match the definition of the ERGM found in equation (1).

RC: ***In the main text, the authors pointed out thirteen exogenous variables widely used for ERGMs. However, in the real data analysis, it was reduced only to stars and triangles***

AR: The 9 other endogenous variables do not require an input parameter and thus do not require algorithm 1. For example let us say we have an observed network with kstar,esp,nsp and dsp upper bounds of 2. Then our initial set of endogenous variables will will consiste of esp(2), nsp(2), dsp(2), kstar(2), triangle,

isolates, sociality, degree cross product, degree popularity, geometrically weighted edgewise shared partners (gwesp), geometrically weighted non-edgewise shared partners (gwnsp), geometrically weighted dyad-wise shared partners (gwdsp) and geometrically weighted degree. Then, algorithm 2 will select the most relevant endogenous variables for the observed network.

RC: ***In general, it is important to clarify how the AIC is computed.***

AR: It was computed via MCMC with initial values obtained via contrastive divergence. This was clarified in the manuscript. "The AIC is computed via MCMC with starting values obtained via contrastive divergence" found at the end of page 5.

RC: ***I also recommend that the authors include a simulation section in the manuscript. Simulating an exponential model with specific variables and subsequently verifying whether the proposed method correctly selects them would certainly enhance the paper.***

AR: This was done with the faux mesa high school network. This network is simulated by an ERGM (hence why it is called "faux" which means false). The method was able to retrieve the endogenous variables that generated it.

2. Reviewer #2

RC: ***In the section "Definition of ERGMs" (pages 2 and 3), the probabilistic model should be better defined. In Equation 1, the term s(y) (the vector of endogenous variables) is not defined along the text. Further, is it modeled the joint probability or marginal probability?***

AR: The other reviewer had the same confusion. The definition was modified across the paper to be the joint probability.

$$P_\theta(Y = \boldsymbol{y}|\boldsymbol{\theta}, \boldsymbol{x}) = \psi(\boldsymbol{\theta}_1, \boldsymbol{\theta}_2) exp\{\boldsymbol{\theta}_1^T s(\boldsymbol{y}) + \boldsymbol{\theta}_2^T g(\boldsymbol{y}, \boldsymbol{x})\} \quad (2)$$

RC: ***It would be interesting a better explanation about endogenous (depend only on y) and exogenous variables (depend on (y,x)). Since the focus is on endogenous variables selection, maybe the definitions, examples and/or interpretations of some endogenous variables such as kstar, triangle, etc should be added***

AR: A paragraph explaining the purpose of endogenous variables and their relation to social networks was added. An interpretation for the triangle term was added.

"These endogenous variables represent characteristics of social networks such as reciprocity, transitivity and centrality. For example, the triangle term measures the transitivity of a network. If a triangle term is included in an ERGM with positive regression coefficient it means that transitivity is a key feature of the observed network "

RC: ***In page 2, the sentence "Inspired by generalized linear models..." is written twice.***

AR: sentence was removed.

RC: ***The equations should be cited along the text. For example, in page 5, the term "... as shown below" should be changed by "...as shown in Equation 2".***

AR: Thanks, the equations are now cited along the text.

RC: ***The notation N is used as the number of nodes but also the number of endogenous variables after the application of Algorithm 1. Maybe it is better to change one of them to avoid misunderstanding.***

AR: Notation was changed to M across all the paper.

RC: ***How does the procedure deal with overfitting? Does the Algorithm 3 try to avoid it in any way? In the same algorithm, does the threshold equals to 4 of step 4 (model exclusion) have any empirical or theoretical justification?***

AR: Algorithm 3 does not handle overfitting. The restriction of having at most 2 endogenous variables in an ERGM handles over fitting. The threshold 4 is a tuning parameter and was found empirically. It means that on average this ERGM produced four times the amount of edges than the observed network (indicating degeneracy). One can increase this number but will run the risk of obtaining degenerate ERGMs.

RC: • ***In the section of "Numerical Studies", it would be interesting to have some discussion or results about the application of Algorithm 1 in the networks. Maybe some table or comments about the obtained upper bounds of the endogenous variables (kstar, esp, etc).***

AR: A table was added that contains the upper bounds of the endogenous variables.
